# SHP-2 Mediates *Cryptosporidium parvum* Infectivity in Human Intestinal Epithelial Cells

**DOI:** 10.1371/journal.pone.0142219

**Published:** 2015-11-10

**Authors:** Eunice A. Varughese, Susan Kasper, Emily M. Anneken, Jagjit S. Yadav

**Affiliations:** 1 Division of Environmental Genetics and Molecular Toxicology, Department of Environmental Health, University of Cincinnati College of Medicine, Cincinnati, Ohio, United States of America; 2 National Exposure Research Laboratory, United States Environmental Protection Agency, Cincinnati, Ohio, United States of America; University of Maryland, College Park, UNITED STATES

## Abstract

The parasite, *Cryptosporidium parvum*, induces human gastroenteritis through infection of host epithelial cells in the small intestine. During the initial stage of infection, *C*. *parvum* is reported to engage host mechanisms at the host cell-parasite interface to form a parasitophorous vacuole. We determined that upon infection, the larger molecular weight proteins in human small intestinal epithelial host cells (FHs 74 Int) appeared to globally undergo tyrosine dephosphorylation. In parallel, expression of the cytoplasmic protein tyrosine phosphatase Src homology-2 domain-containing phosphatase 2 (SHP-2) increased in a time-dependent manner. SHP-2 co-localized with the *C*. *parvum* sporozoite and this interaction increased the rate of *C*. *parvum* infectivity through SH2-mediated SHP-2 activity. Furthermore, we show that one potential target that SHP-2 acts upon is the focal adhesion protein, paxillin, which undergoes moderate dephosphorylation following infection, with inhibition of SHP-2 rescuing paxillin phosphorylation. Importantly, treatment with an inhibitor to SHP-2 and with an inhibitor to paxillin and Src family kinases, effectively decreased the multiplicity of *C*. *parvum* infection in a dose-dependent manner. Thus, our study reveals an important role for SHP-2 in the pathogenesis of *C*. *parvum*. Furthermore, while host proteins can be recruited to participate in the development of the electron dense band at the host cell-parasite interface, our study implies for the first time that SHP-2 appears to be recruited by the *C*. *parvum* sporozoite to regulate infectivity. Taken together, these findings suggest that SHP-2 and its down-stream target paxillin could serve as targets for intervention.

## Introduction

The coccidian protozoan parasite, *Cryptosporidium parvum*, is a major public health concern known to cause outbreaks of gastroenteritis (cryptosporidiosis) worldwide [1]. Based on morbidity, mortality, and economic burden data, the World Health Organization (WHO) has listed cryptosporidiosis as a neglected zoonotic disease in urgent need of renewed research efforts for the prevention of infections in humans [2]. When ingested, the *Cryptosporidium* oocyst undergoes excystation in the small intestine and causes the release of four sporozoites. Upon infection, the parasitic sporozoite utilizes *Cryptosporidium* p30, a galactose/N-acetylgalactosamine-specific lectin (Gal/GalNAc lectin) to bind to the host cell [3]. The sporozoite recruits host cell factors to form a parasitophorous vacuole located inside the apical cell membrane, but still separated from the cytoplasm by an electron-dense membrane. The vacuole provides an extracellular microenvironment for successfully initiating and completing the life cycle of *C*. *parvum* [4].

Given the importance of vacuole formation to *C*. *parvum* infectivity, studies have focused on the host cell-parasite interface in anticipation of discovering mechanisms that could potentially be used to inhibit sporozoite attachment and parasite infection. In the initial stage of infection, the sporozoite engages host cell signaling pathways to recruit host factors for reorganization of the host cell cytoskeleton at the site of attachment. For example, during *C*. *parvum* invasion of biliary epithelial cells, the host tyrosine kinase, c-Src, is activated [5]. The GTPase, CDC42, a key regulator of cytoskeletal reorganization, is also activated in infected biliary epithelial cells to induce actin remodeling, membrane protrusion, and electron dense-band formation. In this process, the downstream effectors of CDC42, Neural Wiskott-Aldrich syndrome protein (N-WASP) and p34-Arc are also recruited to the attachment site [6]. One of the factors that regulate CDC42 activity is frabin, an actin filament (F-actin)-binding protein with GDP/GTP exchange activity. *C*. *parvum* infection induces accumulation and activation of phosphatidylinositol 3-kinase (PI3K) and recruitment of frabin to the attachment site. Furthermore, inhibition of PI3K signaling and/or frabin were shown to inhibit *C*. *parvum* invasion [7, 8]. In AIDS patients, *C*. *parvum* is an opportunistic pathogen. In these patients, expression of integrin α2 (ITGA2), an important receptor involved in cell adhesion, increased following *C*. *parvum* infection. Knockdown of ITGA2 in HCT-8 cells (human illeocecal epithelial cells) reduced the rate of *C*. *parvum* infection [9].

Surprisingly, little is known of the mechanisms by which the host proteins contribute to the formation of the vacuole itself. While tyrosine kinases have been implicated in the formation of the host cell–parasite interface [5, 7], the function of tyrosine phosphatases and their potential role in modulating *C*. *parvum* host interactions remains elusive. Our study for the first time implicates the tyrosine phosphatase Src homology-2 domain-containing phosphatase 2 (SHP-2) in this process. SHP-2, encoded by the human PTPN11 gene, is a ubiquitously expressed protein tyrosine phosphatase that possesses two tandem Src homology (SH2) domains (N-SH2 and C-SH2). In its inactive form, SHP-2 is auto-inhibited due to interactions between the N-terminal SH2 (N-SH2) domain and the catalytic protein phosphatase domain. Binding of pTyr-containing ligands to the SH2 domain disrupts this intramolecular interactions, exposes the tyrosine phosphatase domain, and leads to catalytic activation [10–12]. SHP-2 activating pTyr ligands include growth factor receptors, such as, platelet-derived growth factor receptor, EGF receptor, and the erythropoietin receptor, as well as docking proteins such as paxillin, IRS1, IRS2, and GAB1 [13, 14]. SHP-2 has been shown to regulate the phosphorylation status of focal adhesion proteins, such as paxillin. Paxillin is an adaptor in the integrin pathway, and provides multiple docking sites for signaling molecules in addition to actin binding proteins [15–18].

Previously, we demonstrated that FHs 74 Int cells showed the highest levels of infectivity as compared to the other available cell lines [19]. Using the FHs 74 Int cell type, this study for the first time implicates SHP-2 in the process of *C*. *parvum* infectivity. In FHs 74 Int cells infected with *C*. *parvum*, SHP-2 expression was upregulated for up to 4 hours post infection which resulted in a modest dephosphorylation of the SHP-2 substrate, paxillin. Our results show that the recruitment of the tyrosine phosphatase, SHP-2, is essential for efficient infection and raises the possibility that phosphatases may play an important role in the *C*. *parvum* infection process.

## Materials and Methods

### Mammalian cell lines and *C*. *parvum* strain

FHs 74 Int human small-intestinal epithelial cells (FHs 74 Int) (CCL-241; ATCC, Manassas, VA) were cultured in Hybri-Care Medium (ATCC) with 10% fetal bovine serum (FBS; HyClone, Logan, UT), supplemented with 45 ng/ml of human epidermal growth factor (EGF; Invitrogen, Carlsbad, CA), 1.5 g/L of sodium bicarbonate, 100 U/ml penicillin (Gibco, Gaithersburg, MD), and 100 μg/ml streptomycin (Gibco). Cells were cultured as monolayers in cell culture flasks in 5% CO2 at 37°C.

Viable *C*. *parvum* oocysts (Iowa isolate) were originally purchased from Waterborne Inc. (New Orleans, LA), and continually propagated at the USEPA facility in Cincinnati, OH, using immunosuppressed CF-1 mice. The mice were purchased from Charles River Laboratories, Inc. The animals were allowed to acclimate for 1–3 days, and given food and water *ad libitum*. All procedures in this study were done in compliance with the US EPA Institutional Animal Care and Use Committee regulations. For immunosuppression, the mice were given an alternative day dosing of dexamethazone and tetracycline in the drinking water. After immunosuppression for 7 days, mice were dosed with 10^5^ oocysts, per oral administration using a ball-end dosing needle. Animals were maintained in raised stainless steel mesh grid floors, and their feces were collected every 36 hours. Each fecal collection was processed by sieving and multiple flotations, as previously described [20]. Mice were euthanized using CO_2_ gas, containing at least 5% O_2_. Purified oocysts were stored at 4°C in reagent-grade water containing 100 U/ml penicillin and 100 μg/ml streptomycin. All oocyst lots were used within two months of shedding.

### Antibodies, inhibitors, and reagents

Monoclonal antibodies used were mouse anti-SHP-2 (610621; BD Transduction Laboratories; San Jose, CA) and purified mouse anti-paxillin (612405; BD Transduction Laboratories). Polyclonal antibodies were rabbit anti-phospho-paxillin (p-paxillin) which detects paxillin only when phosphorylated at the tyrosine 118 residue (Tyr 118) (#2541; Cell Signaling, Danvers, MA). Secondary antibodies conjugated to Cy3 or Cy5 were from GE Healthcare. Inhibitors used were against SHP-2: SHP 2 PTPase Inhibitor (NSC-87877), (565851; EMD Millipore, Billerica, MA); against paxillin: Saracatinib (AZD0530) (S1006; Selleckchem, Houston, TX) [21]; and against phosphatases was PTP inhibitor X sodium orthovanadate (567540; EMD Millipore). Cytotoxicity and viability assays were performed on the cells using all the concentrations used for the inhibitors to confirm that there were no cytotoxic effects or reduced cell viability compared to the controls.

### Infection of cell monolayers

*C*. *parvum* oocysts were prepared for infection as previously described [19]. A multiplicity of infection (moi) of ~10:1 was used for the entry experiments assayed by fluorescence microscopy and Western blotting. For infection, oocysts were transferred to 2 mL Flat Top microcentrifuge tubes (Fisher Scientific, Pittsburgh, PA) and centrifuged at 4000 x g, for 3 min at 4°C. Oocysts were excysted by pretreatment in acidified (pH 2.0) and pre-warmed (37°C) 1X Hank’s Balanced Salt Solution (HBSS) (Gibco) for 10 min at 37°C, followed by centrifugation and incubation with pre-warmed (37°C) non-acidified 1X HBSS for 10 min at 37°C. After washing by centrifugation, the oocysts were resuspended in excystation medium, consisting of pre-warmed (37°C) RPMI 1640 medium with L-glutamine (Gibco) containing 10% FBS. The oocysts were then placed on the monolayer for infection.

For experiments using immunofluorescence assays, approximately 6 x 10^5^ FHs 74 Int cells were seeded onto 18 mm round coverslips into each well of a 12-well Costar tissue culture plate (Corning, Inc., Corning, NY), and cultured for 24 h. For experiments using protein expression assays, cells were cultured in 150 cm^2^ peel-back Techno Plastic Products (TPP) cell culture flasks for 24 h. Prior to treatment with inhibitor or oocysts, all cells were serum- and EGF-deprived overnight. Cells were stimulated with specific inhibitor, vehicle, or media (negative control) for 2 h prior to inoculation with oocysts. Prior to inoculation onto the monolayer, oocysts were pre-treated for excystation, as described above. Cells were cultured under standard conditions at 37°C and 5% CO_2_ during the infection process and treatment with inhibitors.

### Immunofluorescence analysis of entry

Infection was stopped at 2.5 h post-inoculation by removing the culture medium. Non-adherent sporozoites and oocysts were removed by washing the infected and non-infected (control) cells three times with cold 1X HBSS. Cells were then fixed onto the coverslip with 4% paraformaldehyde for 10 min, permeabilized with 0.25% Triton X-100 (Sigma, St. Louis, MO) for 10 min, and blocked using fresh 1% bovine serum albumin (BSA) in 1X HBSS. Intracellular parasites were labeled with biotin-conjugated *Vicia villosa* lectin (VVL) (Vector Laboratories, Burlingame, CA) as previously described [22] at a 1:333 dilution of VVL in 1% BSA. After 1 h incubation, the coverslips were washed several times, followed by a second 1 h incubation with Cy5-conjugated streptavidin. For staining of SHP-2, the slides were then washed again several times and stained with 1:500 dilution of antibodies to SHP-2, followed by more washes and a fourth 1 h incubation with Cy3-conjugated anti-mouse IgG antibodies.

An LSM 700 confocal microscope (Zeiss, Jena, Germany), was used to detect Cy3 and Cy5 emissions (492/510 nm and 650/670 nm respectively). Serial Z-stack images (0.45 microns thick) were captured and Zen 2010 software was employed to compile and generate the 3D images. The Cy5 and Cy3 images (artificially colored in red and green, respectively) were overlaid, resulting in *C*. *parvum* appearing red, host cell protein appearing green, and any co-localization appearing yellow. Stained slides were screened for the presence of infection at several magnification levels including 400X, 630X, and 1000X. For quantification of parasites, the number of intracellular *C*. *parvum* were counted per field of view at 200X.

### Protein purification and Western blot

After 2–6 h of infection time, flasks were washed three times with cold 1XHBSS to remove non-adherent sporozoites and oocysts. Cells were lysed and solubilized using RIPA lysis and extraction buffer (Life Technologies, Inc., Carlsbad, CA) supplemented with Halt protease and phosphatase inhibitor cocktail (100X) (Life Technologies, Inc). Lysates were clarified by centrifugation at 13,000xg for 10 min at -9˚C, followed by quantification using BCA Protein Kit (Life Technologies, Inc.). Proteins were resolved by SDS-PAGE using 4–15% Mini-PROTEAN® TGX™ precast gels (BioRad, Hercules, CA), and transferred onto a low fluorescence PVDF membrane. The membranes were then blocked by incubation in Odyssey Blocking Buffer (Li-Cor, Inc., Lincoln, NE) for 1 h followed by incubation with the appropriate antibody diluted in the blocking buffer overnight. Detection was done using ECL Plex Cy3 or Cy5 conjugate secondary antibodies (GE Healthcare, Noblesville, IN), followed by excitation with a Typhoon Fluorescence Imager (GE Healthcare). Generated images were quantified using Image J software. Experiments were performed on at least three separate occasions.

### SH2 domain profiling assay

An SH2 domain-based receptor tyrosine kinase (RTK) profiling kit (Signosis, Inc., Santa Clara, CA) was used to detect active SHP-2. The kit contained microtiter wells coated with the specific SH2 domains of several proteins, including SHP-2 (referred to as PTPN11 in this kit). Using an ELISA format, protein lysates with and without *C*. *parvum* infection were exposed to the immobilized proteins. Bound proteins were labeled using biotin-conjugated pTyr antibodies, followed by streptavidin-conjugated HRP for detection. Absorbance was measured spectrophotometrically at 450nm.

### Statistics

The densitometry analyses of the Western blots are representative of three independent experiments. One-way ANOVA was used as a computation to test various means for equality, using a significance level of 0.05. In cases where the difference between all the means was found to be significant, a post-hoc analysis was done to do a pairwise comparison of the different means. For post-hoc analysis, the Bonferroni Multiple Comparisons Procedure was used and significance was set at either p<0.02 or p<0.05, as specified.

## Results

### *C*. *parvum* infection promotes activation of phosphatases

**Fig 1:**
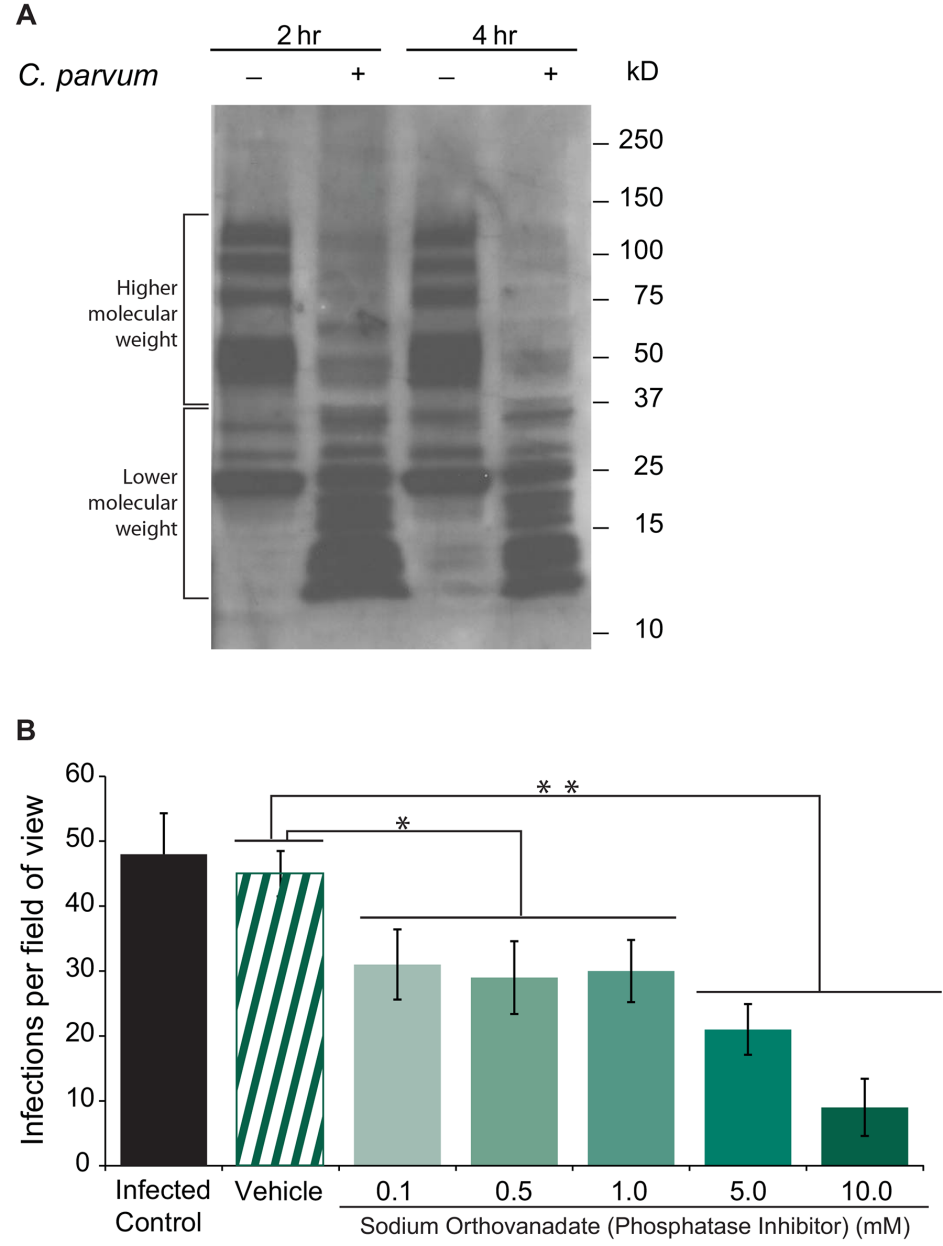
Phosphatase activity is needed for *C*. *parvum* infectivity. A) phospho-Tyrosine blot analysis using whole cell lysates from *C*. *parvum* infected FHs 74 Int cells at 2h and 4 h post infection. Tyrosine-phosphorylated proteins decrease at higher molecular weights upon infection. B) Treatment of FHs 74 Int cells with the phosphatase inhibitor, sodium orthovanadate significantly decreases *C*. *parvum* infectivity. The number of infections per field of view (at 200X magnification) are represented on the y-axis. Values are means ±SD for three independent experiments. Statistical significance criteria set at: *p<0.05, **p<0.02.

The role of phosphatase activity during *C*. *parvum* infection was investigated in two different ways. One mechanism was to evaluate changes in total concentrations of tyrosine phosphorylated proteins before and after *C*. *parvum* infection. Here, we collected total whole cell lysates of non-infected and infected cells 2 h and 4 h after infection. Immobilization of these proteins and immuno-blotting with antibody to phospho-tyrosine revealed that after infection, proteins of higher molecular weights of 125kD to 37kD were less phosphorylated after exposure to *C*. *parvum* (Fig 1A). Image density analysis reveals that in this higher molecular weight range, there is a 37% decrease in tyrosine phosphorylated proteins at 2 h, and a 70% decrease at 4 h, suggesting that tyrosine dephosphorylation is a progressive part of the *C*. *parvum* infection process, and continues up to 4 h after infection. Another mechanism to evaluate phosphatase activity was to determine the effects of the phosphatase inhibitor, sodium orthovanadate on *C*. *parvum* infectivity. When comparing host cells exposed to the phosphatase inhibitor and host cells without the phosphatase inhibitor, we observed that *C*. *parvum* infection sites per field of view decreased significantly (p<0.05) for cells with the inhibitor, even at concentrations as low as 0.1 mM (Fig 1B). The higher doses of phosphatase inhibitor of 5.0 and 10.0 mM resulted in a more significant decrease of infection sites compared to vehicle control (p<0.02), by approximately 47% and 81%, respectively.

### The tyrosine phosphatase SHP-2 plays a role in *C*. *parvum* infection

**Fig 2:**
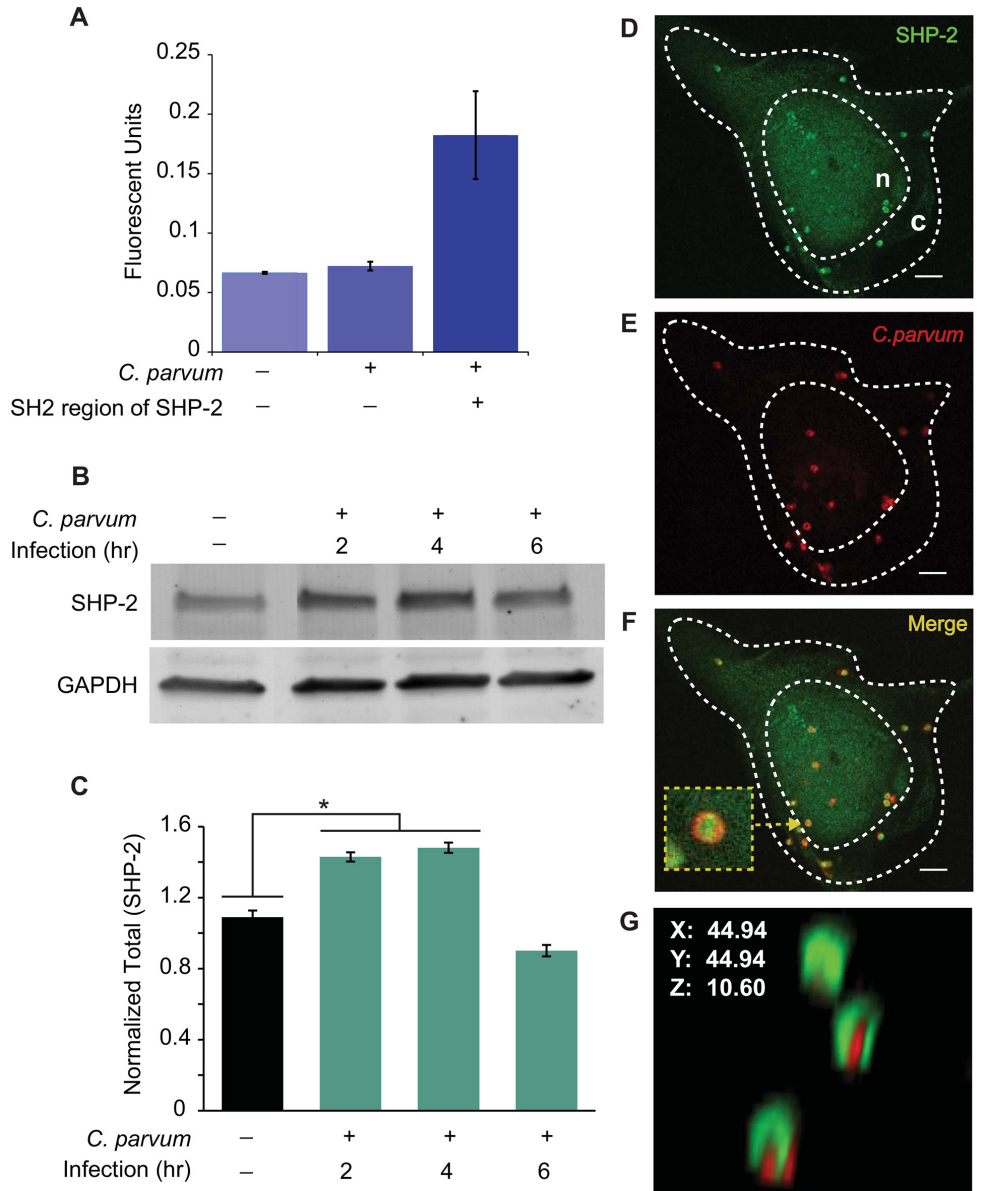
Upon *C*. *parvum* infection, SHP-2 activity and expression increase; and SHP-2 co-localizes with the *C*. *parvum* sporozoite. A) Active binding partners specific to the SH2 domain of SHP-2 increases after 2 h of *C*. *parvum* infection. Values represent mean ±SD for two independent experiments. B) *C*. *parvum* infection upregulates SHP-2 expression at 2h and 4 h post-infection, and levels decrease to pre-infection levels by 6 h. C) Densitometry of Western blots from three independent experiments reflect normalized fluorescence intensity units of SHP-2 expression using GAPDH as a loading control. Data represented are means ±SD for three independent experiments. Statistical significance criteria set at: *p<0.05. D-F) Single confocal microscopic images demonstrating that SHP-2 and the *C*. *parvum* sporozoite co-localize. G) 3D compiled z-stack image of *C*. *parvum* infected FHs 74 Int cells, showing that SHP-2 localizes apically and around the *C*. *parvum* sporozoite. Cy5/Red, *C*. *parvum*; Cy3/green, SHP-2. Scale bar, 10 μm.

SHP-2, a non-receptor tyrosine phosphatase, is normally auto-inhibited, with the N-terminal SH2 domains interacting with its catalytic protein tyrosine phosphatase domain. Complexing of the N-SH2 with a pTyr-containing ligand reduces interaction with the phosphatase domain, causing the enzyme to switch to its active state [14]. To measure the relative increase or decrease of proteins that bind to the SH2 domain of SHP-2, we used an ELISA-based profiling kit, with the SH2 domains of SHP-2 immobilized in a well of a microtiter plate (Fig 2A). Increased levels of absorbance represent increased availability of specific ligand to the SH2 domain of SHP-2. After 2 h of infection, mean absorbance significantly increased (p<0.05) from 0.066 ± 0.01 and 0.07 ± 0.02 in uninfected controls to 0.18 ± 0.04 in infected cells, indicating that *C*. *parvum* infected cells have an increased level of activated proteins that bind to the SH2 domain of SHP-2. Interestingly, total protein levels of SHP-2 also increase significantly at 2 h and 4 h of infection. However, this expression level starts decreasing by 6 h post infection (Fig 2B and 2C), indicating that SHP-2 is a regulated protein during *C*. *parvum* infection and increased levels of the protein is needed during the initial 4 h of infection. This finding coupled with the increased levels of activated binding partners to SHP-2, suggests that SHP-2 may be one of the many phosphatases that play a role in the tyrosine dephosphorylation seen in Fig 1A.

### SHP-2 co-localizes with the sporozoite at the *C*. *parvum* infection site

SHP-2 preferentially localizes in the cytoplasm; however, it has been proposed that the binding of SHP-2 to a pTyr-containing ligand could promote subcellular relocalization of SHP-2 to the cell membrane [13]. Therefore, we determined the cellular localization of SHP-2 after *C*. *parvum* infection. Following infection, bright speckles of SHP-2 appeared on the background of cytoplasmic SHP-2 staining (Fig 2D). In addition, staining of *C*. *parvum* showed that the sporozoites co-localized to the SHP-2 speckles (Fig 2E and 2F). The co-localization was observed at approximately 95% of infections sites. To further characterize SHP-2 and sporozoite co-localization, three-dimensional images were generated by Z-stacking the series of confocal images from just below the basal region of the cell through to just above the apical region of the cell. As seen in Fig 2G, SHP-2 formed a cap over the sporozoite, with finger-like projections extending down the sides of the sporozoite. The specific localization of SHP-2 around the sporozoite suggests that SHP-2 is recruited early in the infection process and may be involved in the initial formation of the parasitophorous vacuole around the parasite.

### SHP-2 activity is required for *C*. *parvum* infectivity

**Fig 3:**
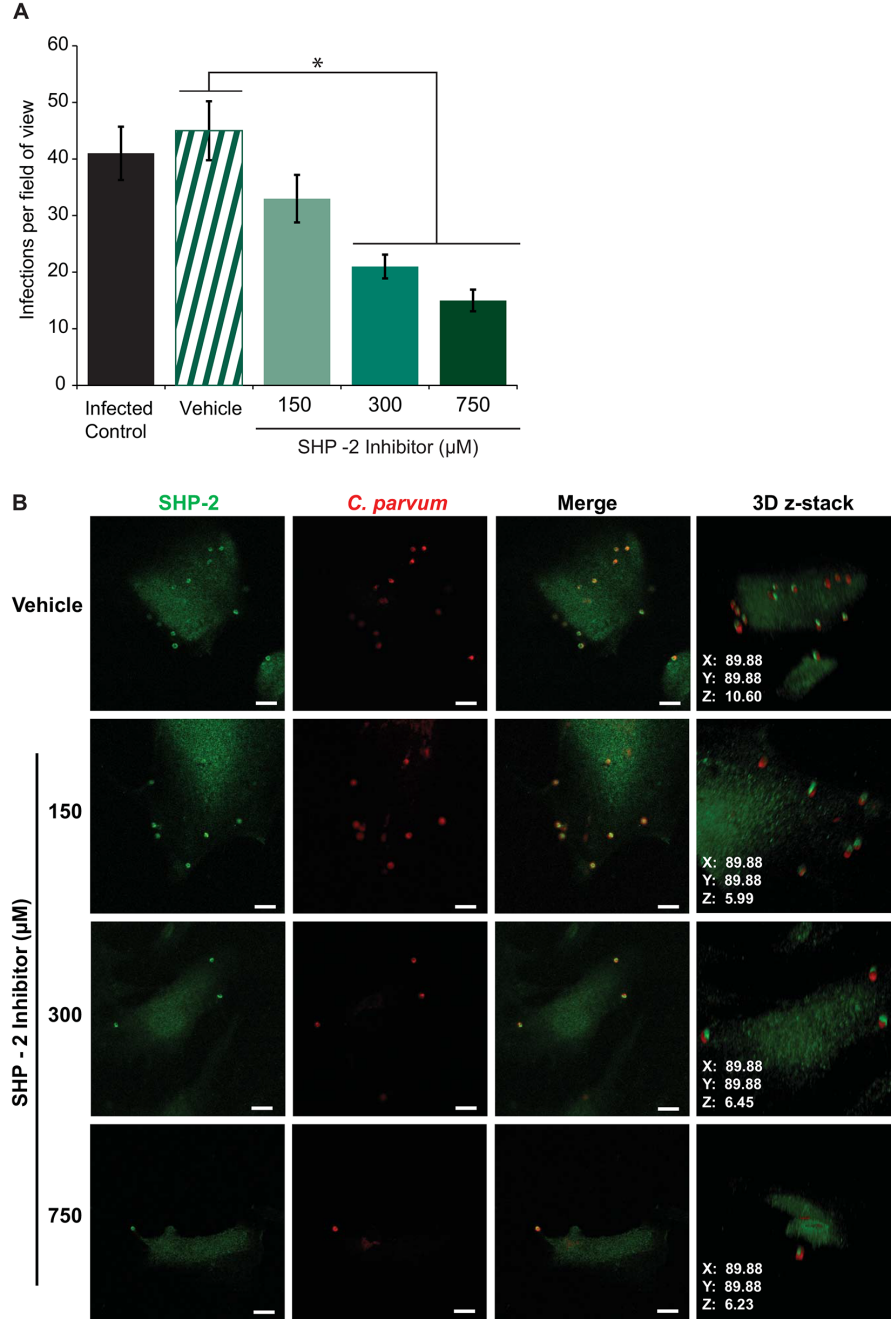
Inhibition of SHP-2 activity decreases *C*. *parvum* infectivity. A) Dose-dependent decrease of Infections per field of view (y-axis) after treatment of FHs 74 Int cells with increasing concentrations of the SHP-2-specific inhibitor, SHP-2 PTPase Inhibitor. Values are means ±SD for three independent experiments. Statistical significance criteria set at: *p<0.05. B) SHP-2 inhibitor treatment does not alter SHP-2/sporozoite co-localization in the few infections sites that still form after SHP-2 PTPase Inhibitor treatment. Cy5/Red, *C*. *parvum*; Cy3/green, SHP-2. Scale bar, 10 μm.

Although we had determined that SHP-2 expression and level of binding partners increased after infection to *C*. *parvum*, we did not know whether activity of SHP-2 is necessary for *C*. *parvum* infection. We examined the need for SHP-2 activity by using a SHP-2 specific chemical inhibitor, SHP-2 PTPase Inhibitor, to block the activity of SHP-2 in the host cell. Inhibitor doses of 300 and 700 μM, showed a significant reduction in the number of infection sites observed per field of view using immunofluorescence microscopy (p<0.05) by 53% and 67%, respectively (Fig 3A). In addition, while the total number of infection sites decreased in a dose-dependent manner, the few infection sites which formed revealed that SHP-2 and the sporozoite remained co-localized even at the highest concentration of inhibitor tested (Fig 3B). The localization of SHP-2 around the parasite and the reduction of infection after exposure to a specific inhibitor of SHP-2, denotes that SHP-2 localization and phosphatase activity play key and necessary roles in the infectivity of *C*. *parvum*.

### Paxillin is moderately dephosphorylated during *C*. *parvum* infection

**Fig 4:**
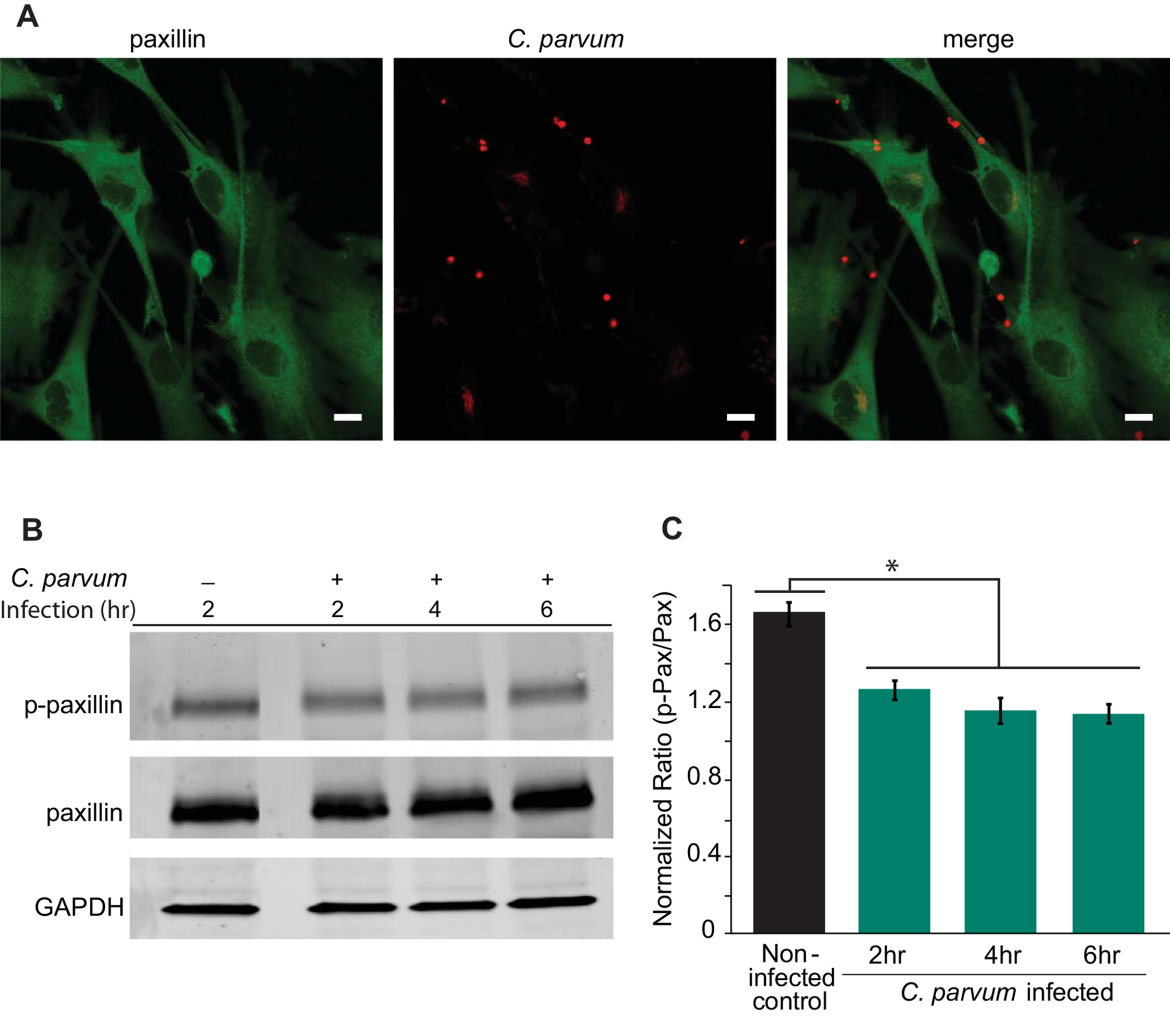
Paxillin, a substrate of SHP-2, does not co-localize with *C*. *parvum* infection site, but is moderately dephosphorylated upon infection. A) Paxillin does not co-localize with the *C*. *parvum* sporozoite. Cy5/Red, *C*. *parvum*; Cy3/green, paxillin. Scale bar, 10 μm. B) Although expression of total paxillin does not change after infection, paxillin is moderately dephosphorylated for up to 6 hours post-infection. C) Densitometry of Western blots from three independent experiments, with normalized fluorescence intensity units of phospho-paxillin expression, using total paxillin as baseline, and GAPDH as a loading control. Data represented are means ±SD for three independent experiments. Statistical significance criteria set at: *p<0.05.

Paxillin, as an adaptor protein and a substrate of SHP-2, contains at least four tyrosine phosphorylation sites that can bind with SH2 domains [23]. Phosphorylation and de-phosphorylation of paxillin at these sites is thought to regulate cell adhesion, among many other cell activities. For example, the changes in the phosphorylation status of paxillin has shown to increase cell migration by decreasing cell adhesion [24, 25]. Although there have been many proteins associated with the phosphorylation for paxillin, there are very few proteins identified that dephosphorylate paxillin. While paxillin is dephosphorylated by SHP-2 [16, 26], the role of paxillin in *C*. *parvum* infection is unknown. Therefore, we determined the cellular localization and phosphorylation status of paxillin with/without *C*. *parvum* infection. After *C*. *parvum* infection, immunofluorescence staining for paxillin demonstrated that it remained localized in the cytoplasm (Fig 4A). Therefore, in contrast to that observed for SHP-2, paxillin did not localize with the sporozoite infection site. However, we did see a moderate, but statistically significant (p<0.05), dephosphorylation of paxillin, at the Tyr118 residue, as early as 2 h post-infection (Fig 4B and 4C). This moderate dephosphorylation continues even up to 6 h post-infection. On the other hand, total paxillin expression remains relatively unchanged up to 6 h. The lack of co-localization suggests that paxillin may not directly interact with *C*. *parvum*, but these observations suggest that moderate dephosphorylation of paxillin may play a role in the continued infection process of the parasite.

### Possible downstream targets of SHP-2 during *C*. *parvum* infection

**Fig 5:**
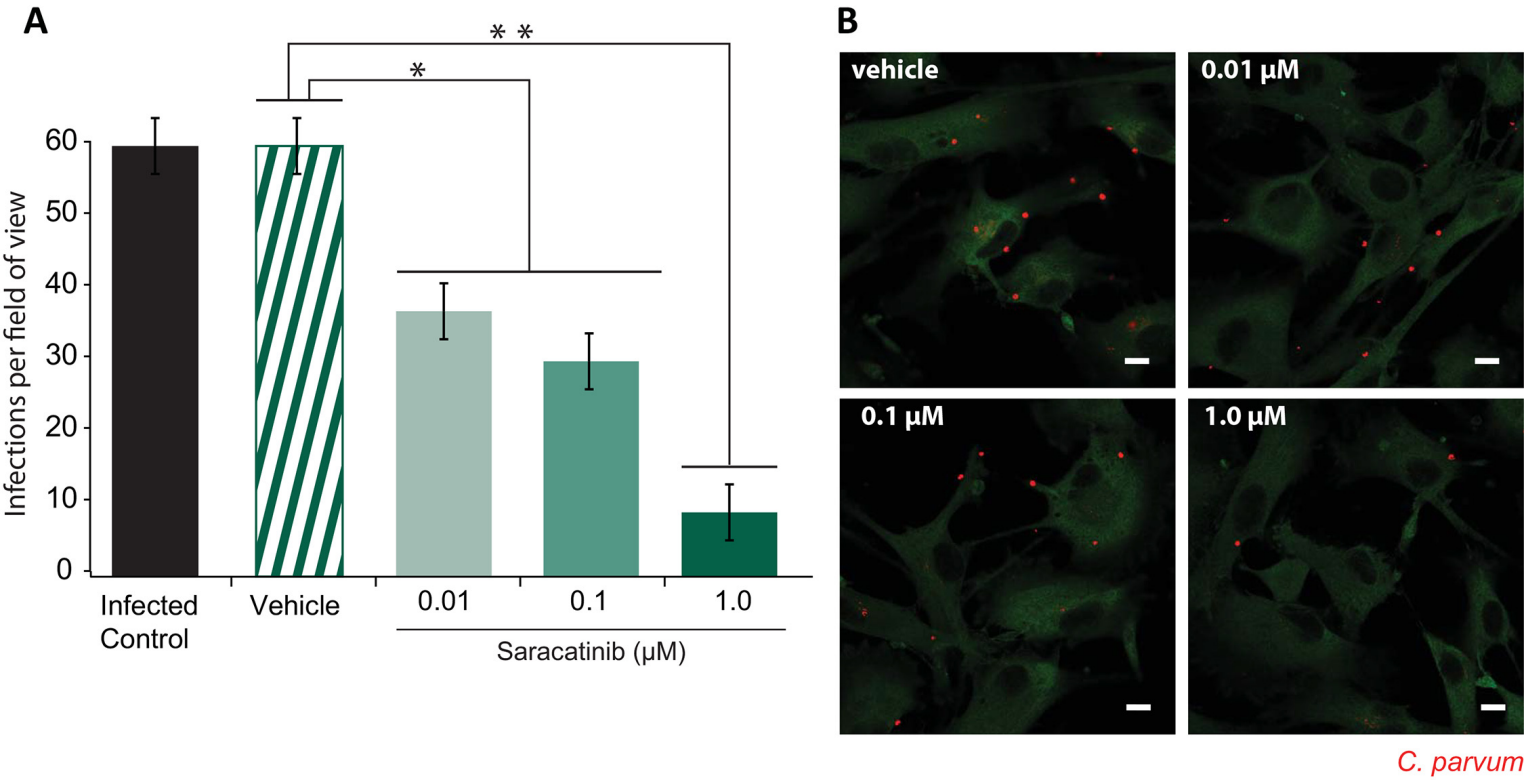
Inhibition of Src kinases and paxillin leads to dose-dependent decreased infection by *C*. *parvum*. A) Number of infections per field of view decrease after exposure of host cells to increased levels of the inhibitor, Saracatinib (AZD0530). Values are means ±SD for three independent experiments. Statistical significance criteria set at: *p<0.05, **p<0.02. B) Merged confocal images of FHs 74 Int cells labeled for paxillin and *C*. *parvum*, show decreased number of infections with increased concentrations of Saracatinib. Red, *C*. *parvum*; green, paxillin. Scale bar, 10 μm.

Since we noticed only a moderate decrease in paxillin phosphorylation, we hypothesized that SHP-2 may be acting on other downstream targets as well. Previous studies have shown that c-Src kinase, a substrate of SHP-2, is necessary for *C*. *parvum* infection [5], and thus we used the inhibitor, Saracatinib, which strongly inhibits Src kinase, and which has been shown to have inhibitory effects on paxillin [21]. Cells were treated with Saracatinib prior to infection, and infection patterns were monitored after 2 h (Fig 5A and 5B). Not only did low doses of the inhibitor significantly decrease infections per field of view (p<0.05), even a relatively low dose of 1.0 μM, reduced *C*. *parvum* infection sites by 87% (p<0.02). In addition, immunofluorescence staining confirmed the decrease in *C*. *parvum* infection (Fig 5B). Together, these observations imply that members of the Src family, possibly other than c-Src, may play a role in *C*. *parvum* infectivity, and that paxillin may also be a factor in infection.

**Fig 6:**
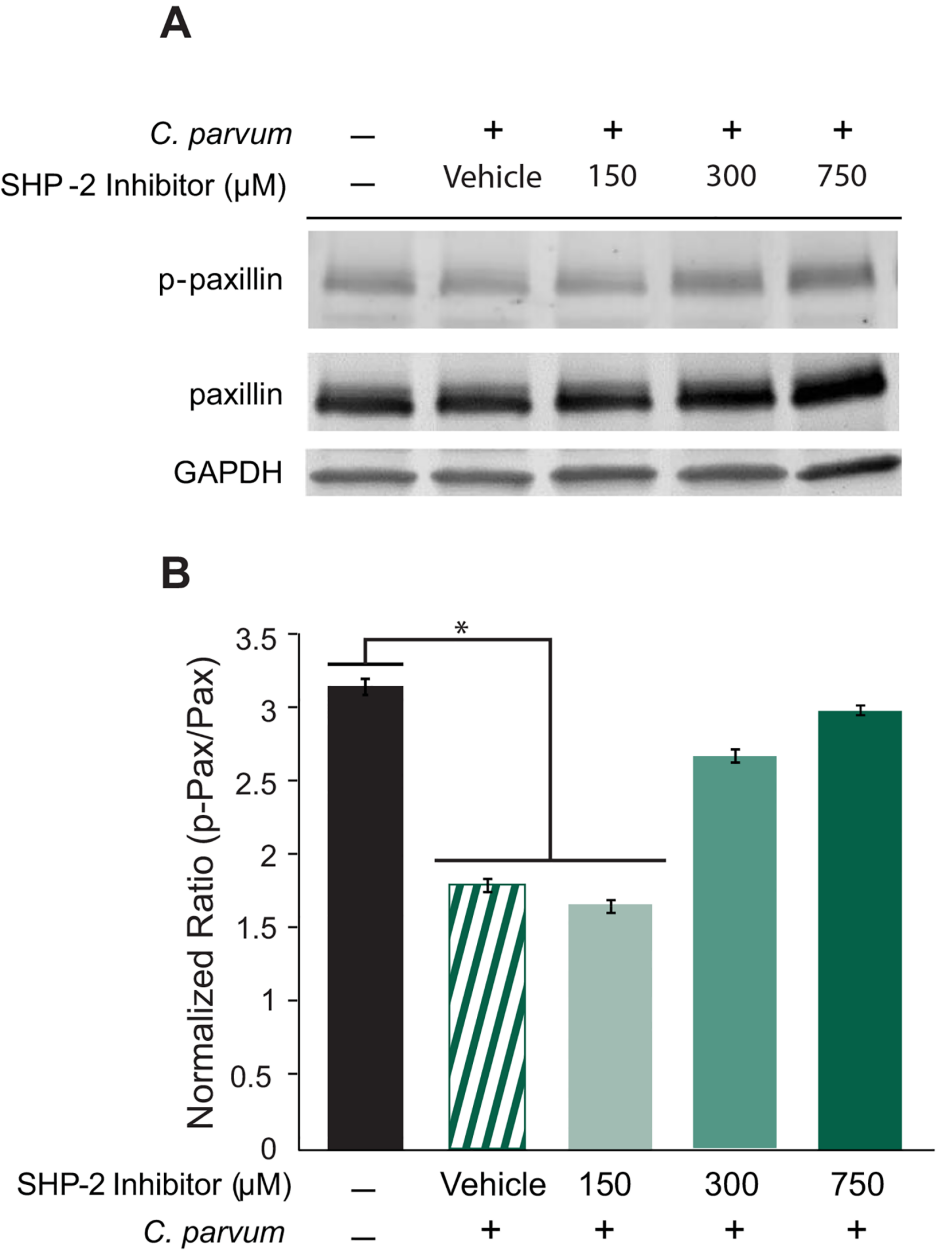
Inhibition of SHP-2 activity promotes paxillin phosphorylation. A) Paxillin phosphorylation levels increase in the presence of 300 and 700 μM SHP-2 PTPase Inhibitor. B) Densitometry of Western blots from three independent experiments, showing normalized fluorescence intensity units of phospho-paxillin expression, using total paxillin as baseline, and GAPDH as a loading control. Data represented are means ±SD for three independent experiments. Statistical significance criteria set at: *p<0.05.

Lastly, the role of SHP-2 in the dephosphorylation of paxillin was investigated. Because SHP-2 has been shown to cause dephosphorylation of paxillin, either directly or indirectly [13, 14], it was hypothesized that exposure of host cells to SHP-2 inhibitor may cause a reversal of the phosphorylation status of paxillin. After exposure of host cells to various levels of SHP-2 inhibitor, we infected the host cells with *C*. *parvum* to see at which doses if any, a change was seen in phosphorylation status (Fig 6A and 6B). After infection, paxillin was moderately, but significantly dephosphorylated for the infected vehicle control, as expected, and the 150 μM concentration of the inhibitor (p<0.5). However in the presence of higher doses of SHP-2 PTPase Inhibitor (300 and 750 μM), paxillin phosphorylation levels increased moderately, close to the state observed in the uninfected controls, confirming that SHP-2 activity may have a moderate effect on the phosphorylation status of paxillin, following *C*. *parvum* infection. Since the change to phosphorylation in paxillin is moderate, SHP-2 may be impacting other proteins more significantly. These results combined with the data regarding decreased infection at the higher levels of SHP-2 inhibitor, suggest that even though paxillin does not co-localize with the infection site, paxillin is moderately dephosphorylated during infection, and that SHP-2 may play a role, whether direct or indirect, in the dephosphorylation of paxillin.

## Discussion

**Fig 7:**
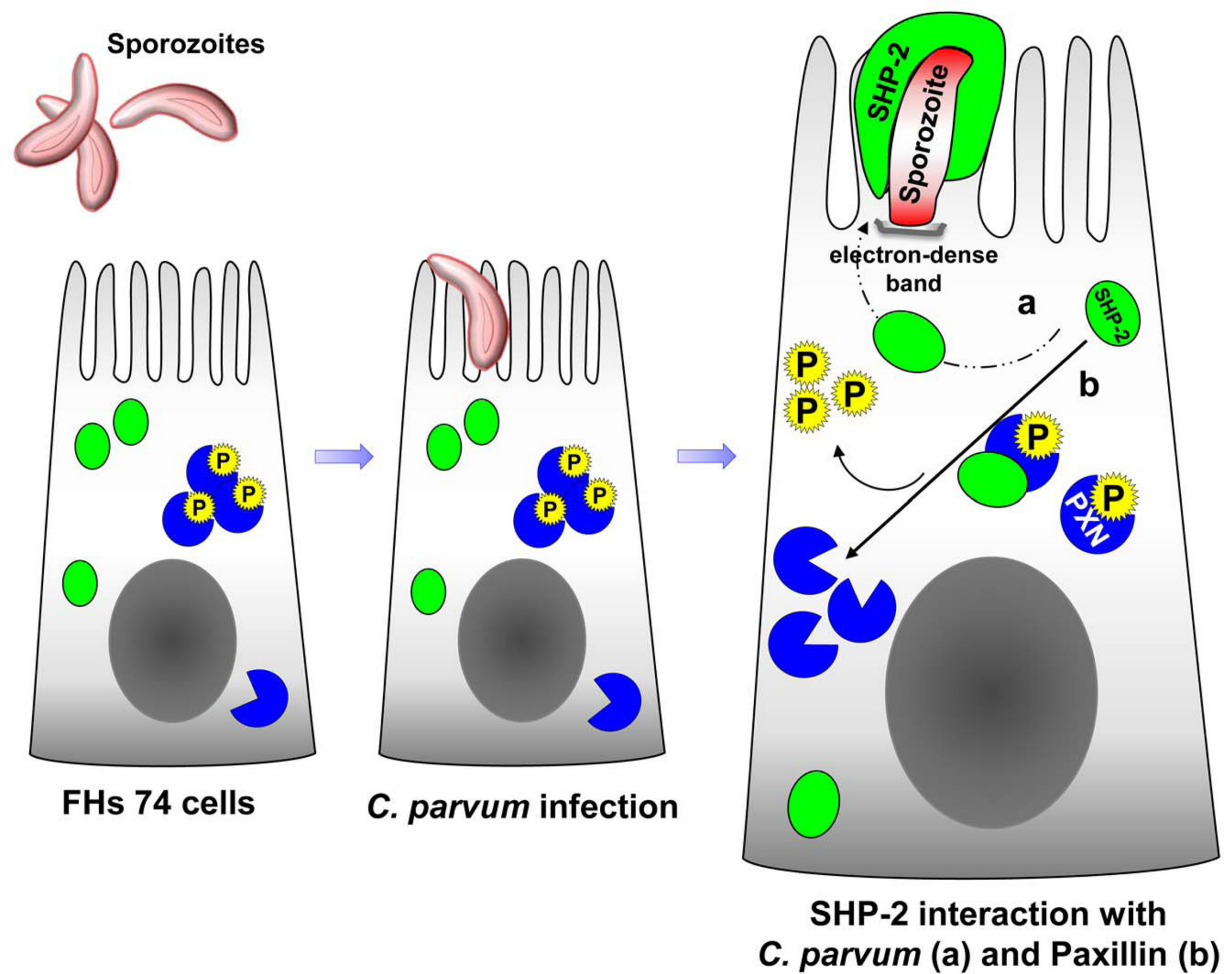
Diagramatic representation summarizing SHP-2-mediated regulation of *C*. *parvum* infectivity. The *C*. *parvum* sporozoite binds to the apical surface of intestinal cells. Upon *C*. *parvum* infection, SHP-2 co-localizes with the *C*. *parvum* sporozoite, forming a cap over the sporozoite with finger-like projects extending down the sides of the parasite. This interaction increases the rate of *C*. *parvum* infectivity through SH2-mediated SHP-2 activity. Whether the interaction of SHP-2 with the sporozoite also participates in forming the parasitophorous vacuole remains to be established. In parallel, SHP-2 moderately dephosphorylates cytoplasmic paxillin, and possibly other proteins, to facilitate *C*. *parvum* infection.

In this study, we identify a novel role for the non-receptor protein tyrosine phosphatase, SHP-2, in the infection of *C*. *parvum*, a parasitic human pathogen (summarized in Fig 7). Upon infection, SHP-2 co-localizes with the *C*. *parvum* sporozoite, thereby increasing the rate of *C*. *parvum* infectivity through SH2-mediated SHP-2 activity. Concurrently, SHP-2 dephosphorylates paxillin to facilitate *C*. *parvum* infection. While paxillin does not directly interact with the sporozoite, it may play a role for infectivity through an unknown mechanism. This study lays the groundwork for further investigation of the mechanisms that regulate *C*. *parvum* infection.

A key aspect of this discovery is that SHP-2 localizes at the *C*. *parvum* infection site and that inhibition of this protein affects the ability of this parasite to infect the host cell. *C*. *parvum*, as an apicomplexan parasite, is an intracellular pathogen. The uniqueness of this parasite relies on its ability to remain extracytoplasmic by the formation of an electron dense-membrane below the vacuole [27]. The formation of this vacuole requires the recruitment of several host cell proteins, including actin [28], however this is the first study that shows the role of a phosphatase in the initial infection process of *C*. *parvum*.

The molecular mechanisms of how SHP-2 initially interacts with *C*. *parvum* and what proteins might be involved in the recruitment of SHP-2 to the infection site has yet to be determined and our current efforts are focused on understanding the early developments of this interaction. It is known that microbial pathogens often inject virulence factors into the host cell upon invasion to start the process of infection. For example, the bacterial pathogen, *H*. *pylori*, releases the virulence factor, Cag A, which binds to and activates SHP-2 [29]. *C*. *parvum* attachment results in the release of several dense granules and the migration of proteins known as micronemes during the initial invasion process. Micronemal proteins are important in host cell localization and ligand-receptor attachment in apicomplexan parasites [30]. Even though just a few micronemal proteins have been identified (GP900, TRAP-C1/2, CpSCRP, and ABD), the roles of these proteins on infection of host cells are not fully understood [31, 32]. It is possible that SHP-2 activation and recruitment is stimulated by one of the many *C*. *parvum* micronemal proteins.

Interestingly, SHP-2 has been shown to be a key host cell factor for virulence by other pathogens, namely, *Helicobacter pylori*, Kaposi’s sarcoma-associated herpesvirus, enteropathogenic *Escherichia coli*, and *Streptococcus pneumoniae* [33–37]. SHP-2 is recruited and activated in these infection systems, and for some pathogens, expression levels of SHP-2 are upregulated to promote infection. However, the purpose for recruitment and activation of SHP-2 is pathogen-dependent. For example, *H*. *pylori* recruits SHP-2 during the early infection process, via a virulence protein named CagA [29, 38]. On the other hand, for other pathogens, SHP-2 plays a role in pathobiology [39], and a role for suppressing the host immune response during infection [36]. In this study, we show expression levels of SHP-2 increase within 2 and 4 hours of infection, and decline by 6 hours post-infection, inferring that SHP-2 upregulation is needed during the early time-points of *C*. *parvum* infection. This is corroborated by the evidence that SHP-2 is localized at the apical end and sides of the infection site, indicating that SHP-2 is needed in the initial infection process of the parasite.

Furthermore, we show that SHP-2 may affect the phosphorylation status of paxillin, a focal adhesion protein, during the infection process. We observe moderate, but significant dephosphorylation of paxillin as early as 2 hours post-infection. Paxillin has focal adhesion targeting regions known as LIM domains, actin binding domains, and phosphorylation sites specific for kinases and phosphatases, which allows it to act as a scaffolding protein that binds multiple proteins. In this study, we observed that the moderate dephosphorylation of paxillin at the Tyr 118 residue was rescued in the presence of increased SHP-2 inhibitor. Nevertheless, paxillin may not serve as a direct ligand of SHP-2. Previous studies have shown that Gab1, a pleckstrin-homology domain-containing docking protein, becomes tyrosine phosphorylated upon various intracellular signals and acts as a scaffolding protein by binding to paxillin and SHP-2 and causing the dephosphorylation of paxillin [26]. This theory is consistent with another study that found that the SH2 domains of SHP2 do not bind directly to phospho-peptides derived from paxillin [40]. The ability of SHP-2 to utilize scaffolding proteins may also provide a mechanism for relocalization in the cell.

Interactive partners of SHP-2 during *C*. *parvum* infection in vivo have yet to be determined. Nevertheless, based on this study and other studies relating to *C*. *parvum* pathogenesis, some inferences can be made. Integrins and Src kinases have been shown to interact with SHP-2 [41, 42], and previous work in *C*. *parvum* infectivity has shown that integrin α2 and c-Src are important players in infection [5, 9]. Consistent with these studies, work in our lab has shown that integrin expression is upregulated by almost 100% upon infection and Src family proteins are dephosphorylated in FHs 74 Int cells infected by *C*. *parvum* (data not shown). However, as with paxillin, we do not see any co-localization with integrin, suggesting that these proteins may function in other mechanisms of *C*. *parvum* pathogenesis, such as survival or cell adhesion. For example, upregulation of integrin might be needed for other purposes such as involvement in the multi-molecular complexes that are needed for focal adhesions linking the extracellular matrix (ECM) with the actin cytoskeleton. Indeed one study found that the force-dependent strengthening of integrin-cytoskeleton linkages requires down-regulation of focal complex dynamics by SHP-2 [17]. Another study found that the binding of integrins to the ECM induces tyrosine phosphorylation of SHPS-1 (SHP substrate) and its subsequent association with SHP-2 [41]. The reinforcement of the host cell to the ECM may provide a unique strategy of a pathogen to prevent epithelial cell turnover. Evidence of this phenomena can be seen in the bacterial pathogen *Shigella*, which uses its virulence factor, OspE, to prevent intestinal epithelial cell detachment by targeting integrin linked kinases to affect reinforcement of cell adhesions. Furthermore, upon OspE expression, host cells had decreased paxillin and FAK phosphorylation levels, which prevented intestinal epithelial cell detachment by targeting integrin-linked kinases to reinforce cell adhesions, and reduce epithelial cell turnover rate [43, 44]. A similar mechanism may be occurring with *C*. *parvum* infection, as it is advantageous for the parasite to progress through its stages of infection before epithelial turnover.

Inhibition of SHP-2 is a promising approach to prevent *C*. *parvum*-induced pathobiology, as there are several potent and highly selective inhibitors to SHP-2 [45]. However, since SHP-2 is a widely-expressed cytoplasmic enzyme and involved in multiple cellular pathways, a targeted approach of inhibition to only infected cells would be a more pragmatic approach. Therefore, a better understanding of the initial events in recruitment and activation of SHP-2 is crucial to designing such an approach.

Our studies reveal a critical role for SHP-2 in the pathogenesis of *C*. *parvum*. We demonstrate for the first time that SHP-2 is recruited to the infection site, and necessary for infection. We further show that SHP-2 may play a role in the dephosphorylation of paxillin. More detailed investigations are required to improve the understanding of the regulation and mechanisms of SHP-2 activation, and whether the activation is through host cell proteins or through *C*. *parvum* virulence factors. Although there is still much to be learned about the mechanisms of pathogenesis of *C*. *parvum*, we have now taken the first steps to show that SHP-2 plays a pivotal role in *C*. *parvum* infection.
